# The gastric H,K-ATPase in stria vascularis contributes to pH regulation of cochlear endolymph but not to K secretion

**DOI:** 10.1186/s12899-016-0024-1

**Published:** 2016-08-11

**Authors:** Hiromitsu Miyazaki, Philine Wangemann, Daniel C. Marcus

**Affiliations:** 1 Department of Anatomy & Physiology, Cellular Biophysics Laboratory, Kansas State University, 228 Coles Hall, Manhattan, KS 66506-5802 USA; 2 Deparment of Anatomy & Physiology, Cell Physiology Laboratory, Kansas State University, 228 Coles Hall, Manhattan, KS 66506-5802 USA; 3 Department of Otolaryngology-Head and Neck Surgery, Tohoku University Graduate School of Medicine, Sendai, 980-8574 Japan

**Keywords:** Acid–base balance, Endolymph, Inner ear, Hydrogen ion secretion, Potassium secretion, Stria vascularis, Ion-selective self-referencing electrode

## Abstract

**Background:**

Disturbance of acid–base balance in the inner ear is known to be associated with hearing loss in a number of conditions including genetic mutations and pharmacologic interventions. Several previous physiologic and immunohistochemical observations lead to proposals of the involvement of acid–base transporters in stria vascularis.

**Results:**

We directly measured acid flux in vitro from the apical side of isolated stria vascularis from adult C57Bl/6 mice with a novel constant-perfusion pH-selective self-referencing probe. Acid efflux that depended on metabolism and ion transport was observed from the apical side of stria vascularis. The acid flux was decreased to about 40 % of control by removal of the metabolic substrate (glucose-free) and by inhibition of the sodium pump (ouabain). The flux was also decreased a) by inhibition of Na,H-exchangers by amiloride, dimethylamiloride (DMA), S3226 and Hoe694, b) by inhibition of Na,2Cl,K-cotransporter (NKCC1) by bumetanide, and c) by the likely inhibition of HCO_3_/anion exchange by DIDS. By contrast, the acid flux was increased by inhibition of gastric H,K-ATPase (SCH28080) but was not affected by an inhibitor of vH-ATPase (bafilomycin).  K flux from stria vascularis was reduced less than 5 % by SCH28080.

**Conclusions:**

These observations suggest that stria vascularis may be an important site of control of cochlear acid–base balance and demonstrate a functional role of several acid–base transporters in stria vascularis, including basolateral H,K-ATPase and apical Na,H-exchange. Previous suggestions that H secretion is mediated by an apical vH-ATPase and that basolateral H,K-ATPase contributes importantly to K secretion in stria vascularis are not supported. These results advance our understanding of inner ear acid–base balance and provide a stronger basis to interpret the etiology of genetic and pharmacologic cochlear dysfunctions that are influenced by endolymphatic pH.

**Electronic supplementary material:**

The online version of this article (doi:10.1186/s12899-016-0024-1) contains supplementary material, which is available to authorized users.

## Background

Disturbance of acid–base balance in the inner ear is known to be associated with hearing loss in a number of conditions including genetic mutations (e.g., Pendred syndrome and hereditary distal renal tubular acidosis [1–4]) and pharmacologic interventions (e.g., acidic-vehicle drug delivery [5, 6]). Homeostasis of endolymphatic (luminal) pH by specific ion transporters in cochlear epithelial cells has been postulated and observed. H^+^ secretion by marginal cells of the stria vascularis was proposed based on observations of immunostaining of vH^+^-ATPase near the apical membrane of the epithelial cells [3, 7]. H^+^,K^+^-ATPase [8] and Na^+^,H^+^ exchangers [9–11] have been immunolocalized to strial marginal cells and both Na^+^,H^+^ exchanger [12] and H^+^-monocarboxylate transporter [13] activity have been observed. A counterbalancing secretion of HCO_3_^−^ by apical pendrin (SLC26A4) in strial spindle cells, spiral prominence and outer sulcus cells is also critical to pH homeostasis and hearing [14].

The goal of the present study was to test the propositions that the stria vascularis secretes H^+^ via vH^+^-ATPase, and that the basolateral H^+^,K^+^-ATPase in strial marginal cells provides a third K^+^ uptake pathway (in addition to the Na^+^,2Cl^−^,K^+^ cotransporter (NKCC1) and Na^+^,K^+^-ATPase) involved in K^+^ secretion. We demonstrate that the stria vascularis can indeed actively secrete H^+^, but via apical Na^+^,H^+^ exchange and not vH^+^-ATPase. The basolateral H^+^,K^+^-ATPase can contribute to this H^+^ secretory flux, but does not mediate a significant basolateral K^+^-uptake in parallel to the other two K^+^-secretory uptake pathways. Further, the apical Na^+^,H^+^ exchanger is poised to aid Na^+^ removal during pathological elevation of endolymphatic Na^+^. These results advance our understanding of inner ear acid–base balance by rejecting some prevailing concepts and by confirming others.

## Methods

### Tissue preparation

Adult C57Bl/6 mice of both genders were deeply anesthetized with 4 % tribromoethanol (0.014 ml/g body wt ip) and sacrificed by decapitation according to a protocol approved by the Kansas State University Institutional Animal Care and Use Committee (#2925). The handling of animals adhered to the ARRIVE guidelines. Stria vascularis (without spiral ligament) was microdissected from the cochlea. Stria vascularis was folded with the apical membrane to the outside of the loop and then mounted in a superfusion chamber on the stage of an inverted microscope and stabilized in the bath with a glass holding pipette.

### Transepithelial hydrogen and potassium fluxes

K^+^ secretion by inner ear epithelia was measured by techniques well-established in this laboratory [15] and extended in this study to also measure H^+^ fluxes. Briefly, glass capillaries were pulled to a tip size of about 4 µm and silanized, filled with reference electrolyte (K^+^: 100 mM KCl in 0.2 % agar; pH: 500 mM KCl +20 mM hepes, pH 7.34 in 0.2 % agar), a short column of liquid ion exchanger (K^+^, potassium ionophore I-cocktail B: 50–100 μm column of Sigma-Fluka #99373; pH, hydrogen ionophore II: 20–30 μm column of Sigma-Fluka #95297) aspirated into the tip and the electrode was connected to an electrometer headstage via a Ag/AgCl wire in contact with the reference electrolyte. The ground was a Ag/AgCl wire connected to the bath via a 1 M KCl agar bridge.

**Fig. 1:**
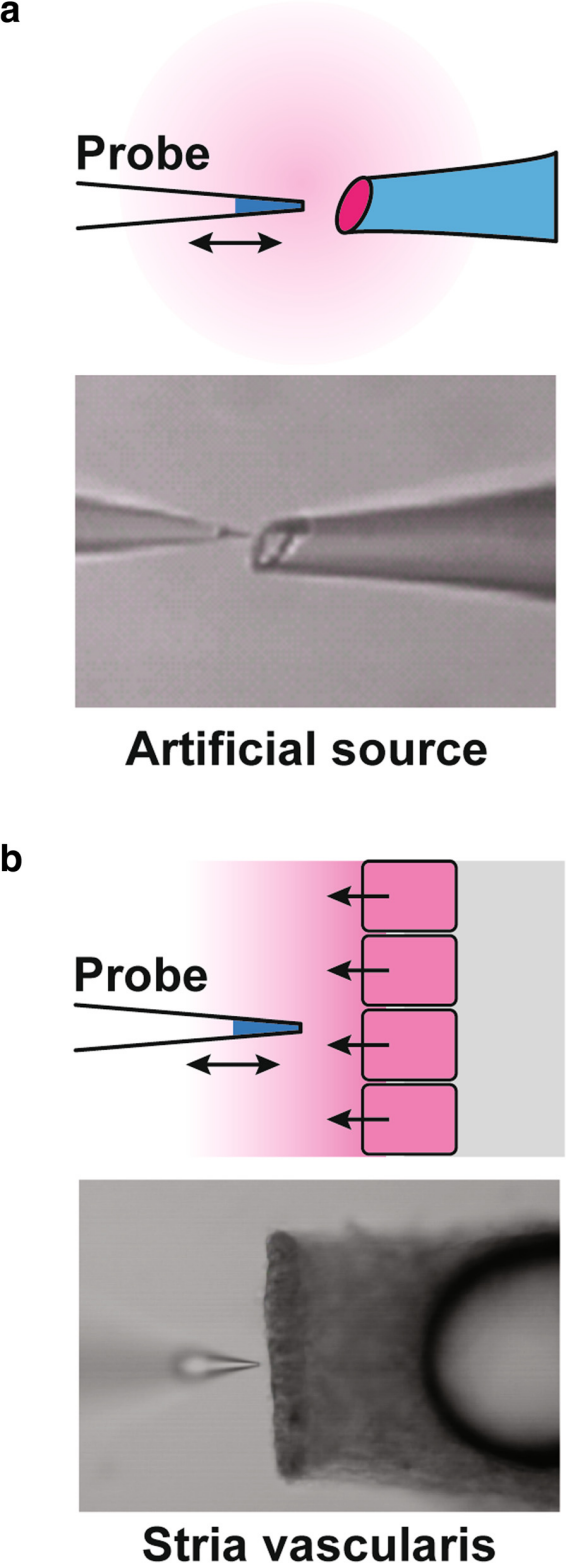
Self-referencing ion-selective electrode method. The electrode (probe) tip oscillates a distance of about 30 μm (*double-headed arrow*). The mean position is first set remote from the source (about 500 μm away; not shown) and the difference signal from the oscillation is taken as zero. The oscillating probe is then set within several μm of the **a** artificial source or **b** epithelium, and the difference signal in the concentration gradient is taken as a relative measure of the ion flux. Changes in ion flux are monitored during bath perfusion of control and experimental solutions. *(Top panels*) diagram; *(Bottom panels*) photomicrographs of artificial and epithelial sources and adjacent probe. Liquid ion exchanger (selective for K^+^ or H^+^) is visible in the tip of the electrode

The electrodes were first tested for a macroscopic slope of 50–60 mV/decade concentration change and then tested for their ability to detect a gradient near an artificial source (Fig. 1a). The artificial source was constructed from a glass pipette (~80 μm tip diameter) filled with 100 mM KCl or 2 mM H_2_SO_4_ solution at pH ~3.6 in 4 % agar, which permitted testing drugs for contributing any additional pH buffering or for interference with the liquid ion exchanger. The tip of the ion-selective electrode was positioned under visual control to within a few micrometers of the apical cell surface or the orifice of the artificial source. The electrode was then oscillated by linear translation stages via computer-controlled stepper motors a distance of 30 μm away from the tissue or source. The electrode signal was recorded at each of these two positions and the difference calculated at intervals of 6.8 s to obtain a representation of the relative flux of K^+^ or H^+^ [16]. This difference voltage was also obtained remote from the source (~500 μm) to establish the zero-flux baseline.

Tissues were fixed to the bottom of a microscope chamber and superfused with solutions at 37 °C. The superfusion solution reached both the apical and basolateral membranes of the marginal cells, since it was determined earlier that dissection of the stria from the spiral ligament compromises the integrity of the basal cell layer [17]. H^+^ flux was measured (Fig. 1b) in a weakly-buffered perilymph-like solution (below) to which transport inhibitors were added. Perfusion conditions were carefully adjusted to obtain the best compromise between good fluid exchange and maintenance of sufficient unstirred layer to establish a measureable concentration gradient near the tissue. Data are expressed as the difference in voltage of the ion-selective electrode across the 30 μm excursion or as the fraction (in %) of that voltage difference under test conditions compared to the value during the control period.

The bath solutions contained (in mM): 150 NaCl, 1.6 K_2_HPO_4_, 0.4 KH_2_PO_4_, 0.7 CaCl_2_, 1.0 MgCl_2_, 5 glucose, pH 7.4 (by NaOH). Measurements were made at 37 °C. Drugs were either pre-dissolved in DMSO [amiloride (Sigma A7410), HOE694 (3-methylsulfonyl-4-piperidinobenzoyl) guanidine methanesulfonate; gift from Dr. H-J. Lang), S3226 (3-[2-(3-guanidino-2-methyl-3-oxo-propenyl)-5-methy]-N-isopropylidene-2-methyl-acrylamide; gift from Sanofi-Aventis Deutschland GmbH), dimethylamiloride (DMA; Sigma A4562), ouabain (Sigma O3125), bumetanide (Sigma B3023), SCH28080 (Sigma S4443), DIDS (4,4’-Diisothiocyanatostilbene-2,2’-disulfonic acid; Sigma D3514)] and used at a final concentration of < =0.1 % DMSO or dissolved directly in bath solution [bafilomycin A1 (Sigma B1793)]. DMSO 0.1 % was also added to the control solution when it was used to dissolve the drug.

Tissues were superfused with the control bath solution (above) for at least 5 min to stabilize the preparation, followed by a 5-min first measurement period, then a 5 to 10 min experimental treatment period, followed by a second 5-min control period. Thirteen to 18 continuous digital samples were averaged near the end of the period prior to the experimental period and from a stable or early quasi-stable time period, typically 3 to 5 min post solution change. When there was a slow secondary phase to the experimental response, an earlier quasi-stable time period was chosen in order to minimize possible drift in the experiment or changes due to secondary effects from the experimental treatment [see Additional file 1].

Measurements from the experimental period were compared to the value from the prior period (time-control, glucose-free, ouabain, 50–200 μM bumetanide, DIDS, bafilomycin) or to the average of the two control periods when the response was clearly reversible (amiloride and its analogs, SCH28080, 1–10 μM bumetanide). Data are expressed as mean ± sem of the signal in μV or percent of the control, *n* = number of tissue samples [see Additional file 1].

Significance was determined by paired *t*-test and *P* < 0.05 was taken as a significant difference. Concentration-response data for SCH28080 and the NHE transport inhibitors were fitted to Michaelis-Menton kinetics using Origin software (OriginLab, Northampton, MA). The fits were made using the individual data points in order to avoid skewed weighting on the logarithmic scale and the best-fit curve then plotted with the average response at each concentration for clarity.

## Results and discussion

### Strial H^+^ flux is linked to metabolism and Na^+^,K^+^-ATPase activity

**Fig. 2:**
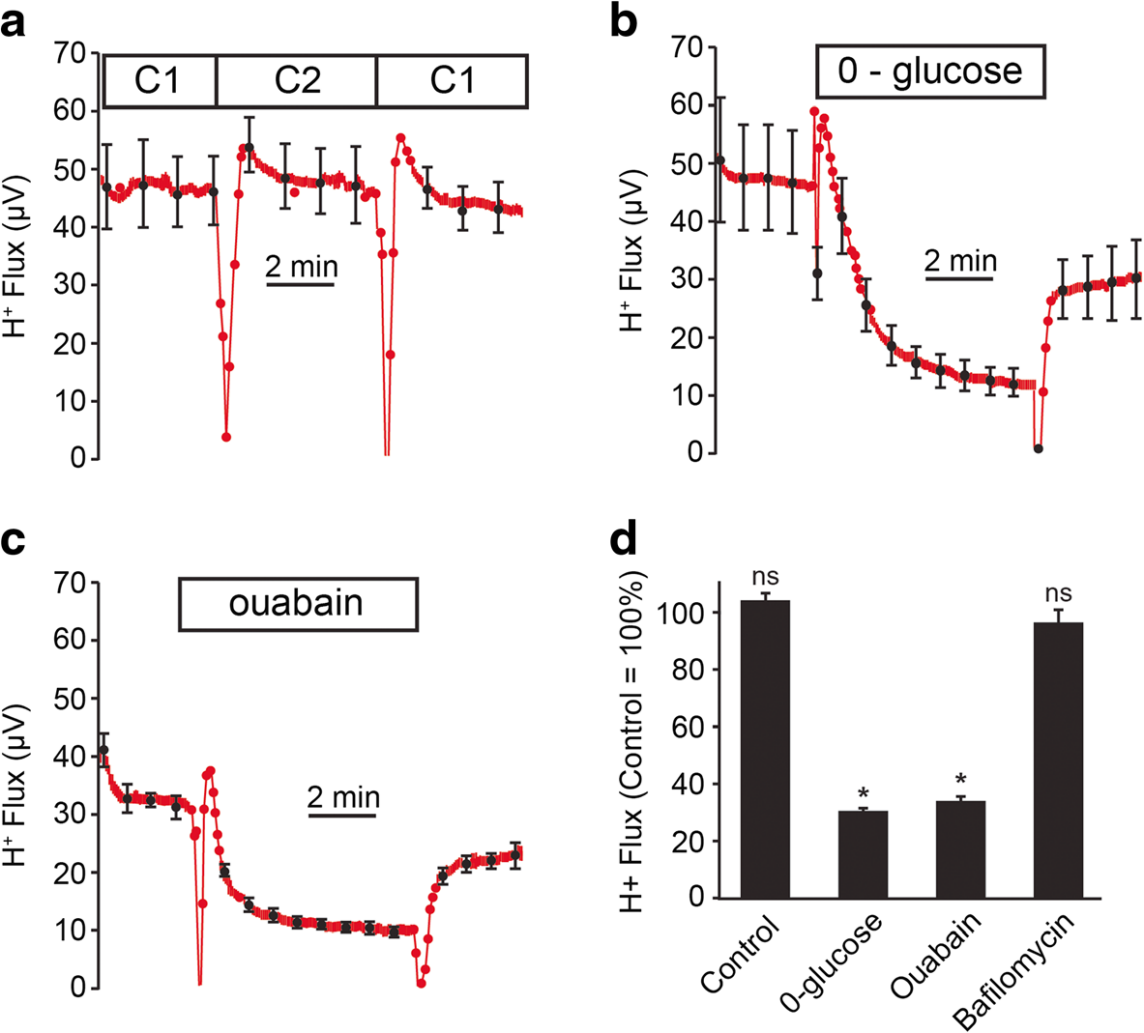
H^+^-flux from stria vascularis; control, glucose-free, ouabain and bafilomycin. **a** Summary traces (*N* = 4) of time-controls during perfusion of control bath solution in the absence of any experimental agents. Boxes labeled C1 and C2 are the perfusion times from two separate reservoirs of bath solution (see Methods for composition). **b** Summary traces (*N* = 3) of glucose-free (0-glucose) perfusion. **c** Summary traces (*N* = 3) of ouabain (1 mM) perfusion. **d** Bar graphs of steady state effects of time-control, glucose-free, ouabain and bafilomycin (1 μM; *N* = 6) perfusion. *, *P* < 0.05; ns, not significant. **a**, **b**, **c** Traces are the vertical averages of N experiments conducted with identical time-course. Standard error bars are indicated only at intervals for clarity; boxes show duration of the experimental period

H^+^ flux was measured near the apical membrane of stria vascularis and the results were not significantly affected by the experimental protocol (Fig. 2a). A substantial H^+^ flux was observed, as indicated by a mean voltage difference at the H^+^-selective microelectrode of 32.6 ± 0.8 μV (*n* = 118) under control conditions. Both glucose-removal and inhibition of the basolateral Na^+^,K^+^-ATPase with ouabain (1 mM) reduced apical H^+^ flux to 37.9 ± 0.6 % (*n* = 3) and to 39.7 ± 2.2 % (*n* = 3) of control values (Fig. 2b, c, d). These results are consistent with the interpretation 1) that strial H^+^ flux requires metabolic energy and 2) that it depends on a transmembrane Na^+^ gradient established by the Na^+^-pump. The remaining ~40 % H^+^ flux after removal of glucose may represent catabolism of other stored metabolic substrates, including glycogen or phosphocreatine, although glycogen stores in the stria vascularis are quite low compared to other inner ear structures [18]. The transport pathway of this remaining H^+^ flux is not yet identified, but may be simple diffusion through H^+^ channels [19] or through other integral membrane proteins [20].

The primary metabolic source of secreted acid is the unusually-high rate of CO_2_ production from glucose, thought to be due to shunting of glycolysis through the hexose monophosphate pathway [21]. Strial metabolism occurs at a remarkably-high rate, similar to that of kidney [22]. The rate of O_2_ consumption decreases by about half when the Na^+^-pump is blocked by ouabain [22]. The coupling of O_2_ respiration rate to CO_2_ production with a respiratory quotient of 1.2 [21] predicts a drop of H^+^ efflux by ouabain of a little more than half, which is consistent with our observation (above).

### Strial H^+^ flux is not carried by an apical vH^+^-ATPase

The apical H^+^ flux in the presence of 1 μM bafilomycin A1, a concentration that would produce a near maximal inhibition of H^+^-ATPase, was not significantly different than under control conditions (Fig. 2d). This result demonstrates that H^+^ flux is not mediated via an apical vH^+^-ATPase and suggests that the immunostained vH^+^-ATPase near the apical membrane of marginal cells [7] is not contributing to H^+^ efflux under our experimental conditions. It is conceivable that the immunostained vH^+^-ATPase is in sub-apical vesicles [23].

### Coupling of strial K^+^ flux to H^+^ flux

**Fig. 3:**
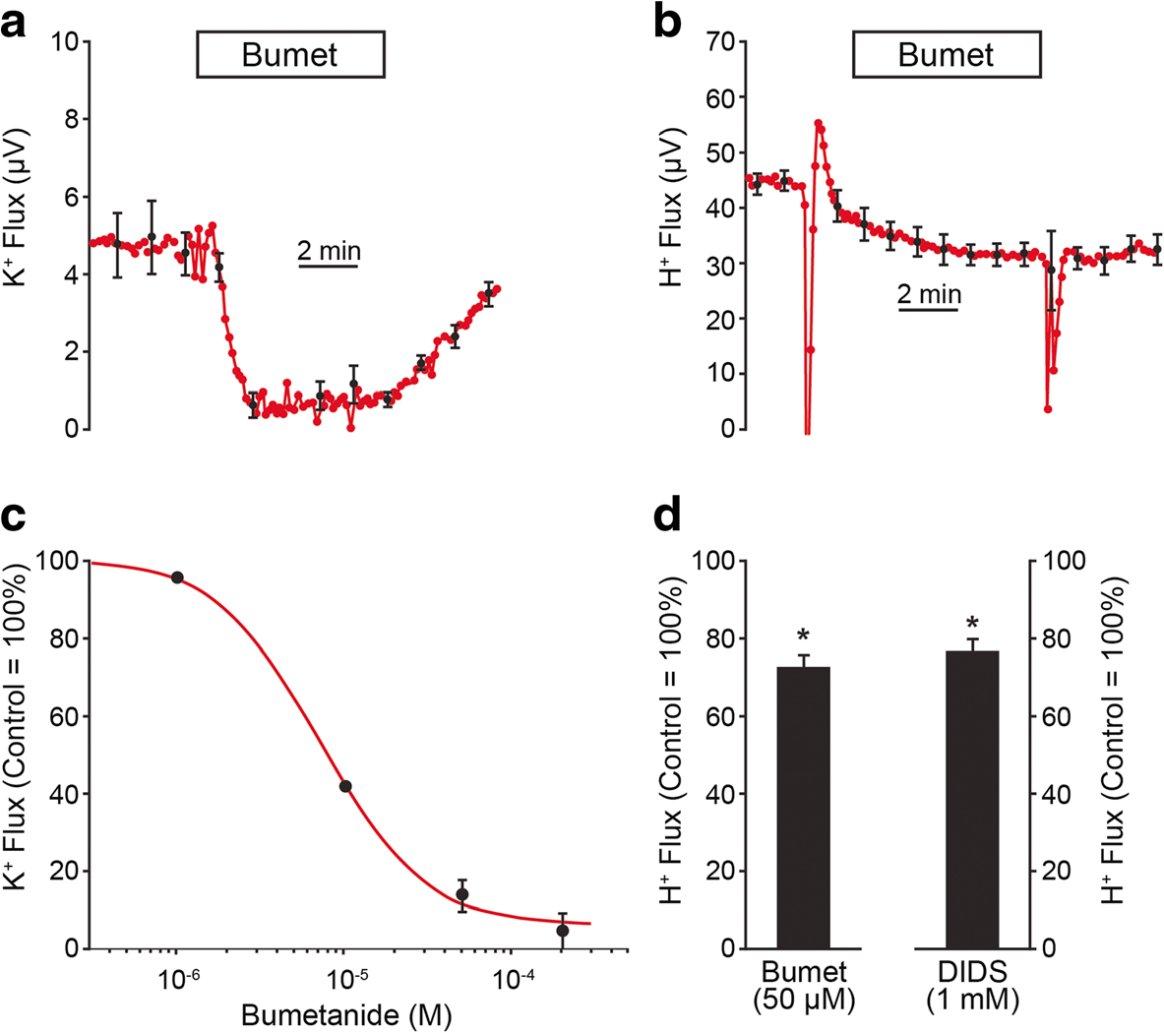
K^+^- and H^+^- fluxes from stria vascularis; bumetanide and DIDS. **a** and **c** K^+^ flux; **b** and **d** H^+^ flux. **a** Summary traces (*N* = 7) of effect of perfusion of bumetanide (50 μM; bumet) on K^+^ flux from stria vascularis. **b** Summary traces (*N* = 6) of effect of perfusion of bumetanide (50 μM) on H^+^ flux from stria vascularis. **c** Concentration-response curve for bumetanide on K^+^ flux from stria vascularis fit by the Michaelis-Menton equation (*N* = 5–6). **d** Bar graph summaries of steady–state effects of bumetanide (50 μM; *N* = 6; Bumet) and DIDS (1 mM; *N* = 6) on H^+^ flux from stria vascularis. *, *P* < 0.05; ns, not significant. **a**, **b**: boxes show duration of the experimental period

The effect of ouabain on H^+^ flux (above) is consistent with a feedback of cellular ion transport to the rate of metabolism needed to power the transport. We evaluated specifically the primary ion transport function of stria (K^+^ secretion) by inhibiting apical K^+^ flux from strial marginal cells by blocking uptake of K^+^ via the basolateral Na^+^,2Cl^−^,K^+^ cotransporter with bumetanide (Fig. 3a, b). The average from all measurements of K^+^ flux under control conditions was 5.6 ± 0.4 μV (*n* = 28). The IC_50_ of bumetanide on K^+^ flux in this series of experiments was 7 × 10^−6^ M, which is consistent with specific inhibition of NKCC1, and is similar to previous findings in vestibular dark cells and strial marginal cells [17, 24, 25]. Indeed, inhibition of K^+^ secretion by bumetanide partially inhibited H^+^ flux (Fig. 3d). This finding is consistent with the view that K^+^ secretion is coupled to metabolic rate and thereby acid production and its subsequent efflux from the cells. The meaning of the observation that H^+^ flux is reduced far more by ouabain than by bumetanide is not clear. These results are consistent with the notion that the Na^+^ pump energizes more processes than K^+^ secretion, such as the Na^+^,H^+^ exchangers (below) and/or other Na^+^-coupled transport [26].

We hypothesized that since reduction of metabolism (either by direct inhibition of metabolism or via inhibition of ion transport) lead to reduced apical H efflux, that stimulation of ion transport was expected to increase apical H efflux. DIDS stimulates K^+^ secretion through potentiation of apical KCNQ1/KCNE1 K^+^ channels [27] as well as its more-widely known inhibitory action on several types of Cl^−^-HCO_3_^−^ transporters, including Slc26a4 [2, 28]. It was expected that if the primary action of DIDS were stimulation of K^+^ secretion, the metabolic rate would be increased and thereby increase the apical H^+^ flux. By contrast, it was observed that DIDS (1 mM), similar to bumetanide, reduced the H^+^ flux from strial marginal cells (Fig. 3d).

This result is inconsistent with our hypothesis and suggests that the primary effect of DIDS may be via its known inhibition of several anion transporters [28]. The drug target is not likely to be the Cl^−^,HCO_3_^−^ exchanger Slc26a4, since Slc26a4 is not expressed in strial marginal cells, and even if HCO_3_^−^ secretion by apical Slc26a4 in remote cells contributed to the net H^+^ flux recorded at the marginal cells, inhibition by DIDS would lead to an increase of net H^+^ flux, and not the observed decrease. One could therefore speculate that there is normally a DIDS-sensitive basolateral HCO_3_^−^ efflux (e.g., via NBCe1 as proposed in the proximal kidney tubule [28]) from strial marginal cells. Inhibition by DIDS of this putative basolateral HCO_3_^−^ efflux would result in an accumulation of intracellular HCO_3_^−^ which would be expected to slow down the hydrating reaction of CO_2_ catalyzed by carbonic anhydrase, thereby reducing the available cytosolic H^+^ and apical H^+^ efflux, as observed. The unreacted CO_2_ would diffuse passively across the basolateral membrane and be carried away in the blood (in vivo) or bath (in vitro). The actual mechanism remains unknown.

### Strial H^+^ flux is carried by apical Na^+^,H^+^ exchangers

**Fig. 4:**
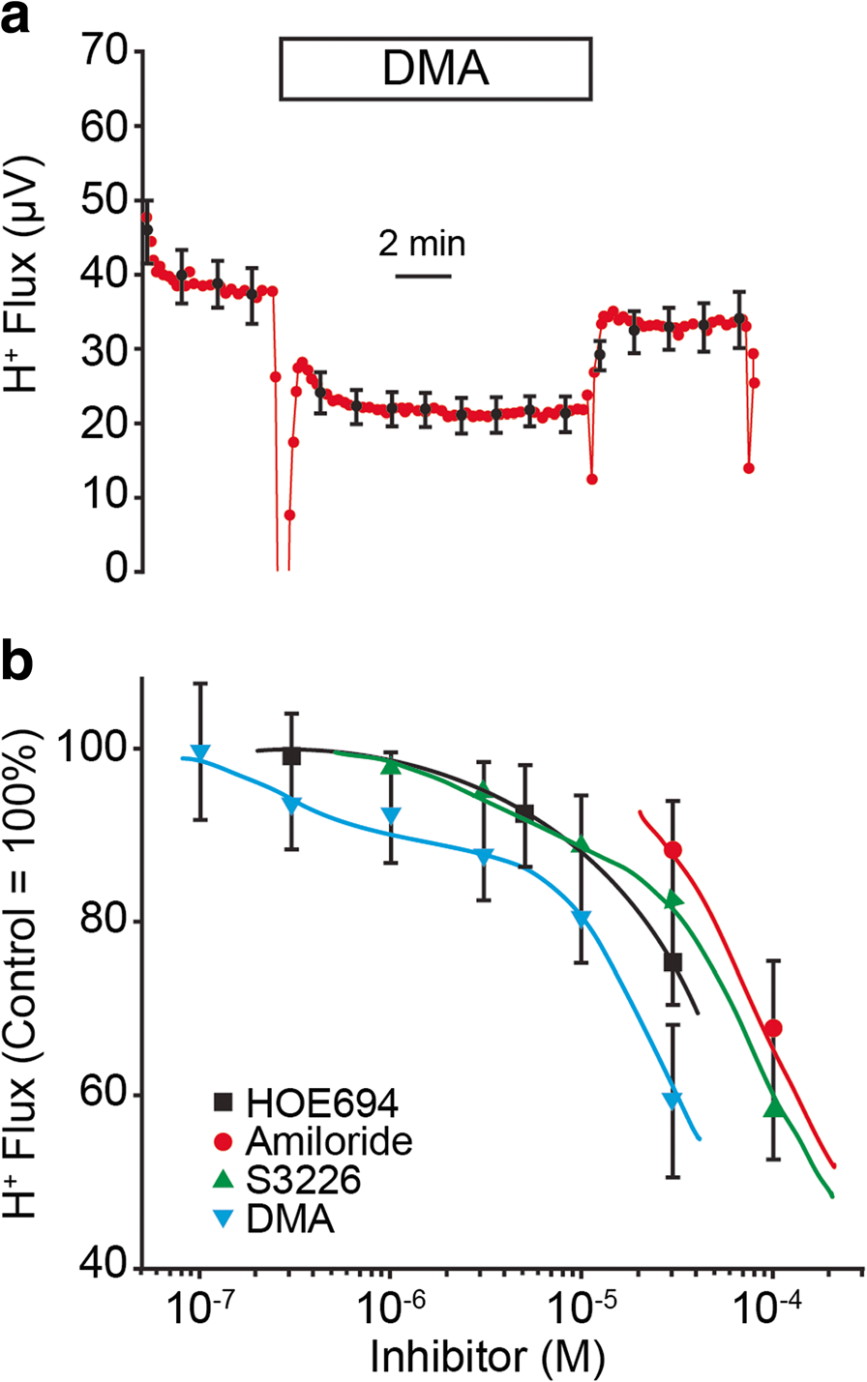
Inhibition by amiloride analogs of strial H^+^ flux from stria vascularis. **a** Summary traces of perfusion of dimethylamiloride (DMA; 30 μM; *N* = 5); box shows the duration of the experimental period. **b** summary family of curves for four Na^+^,H^+^ exchanger inhibitors: HOE694 (*N* = 3–7), amiloride (*N* = 4–6), S3226 (*N* = 3–6) and DMA (*N* = 3–9). Data from DMA and S3226 were fitted with a dual Michaelis-Menton equation consisting of one component that contributes 18 % (putative apical NHEs) and a second component that contributes 82 % (putative metabolic pathway target) of the total response

It was hypothesized that apical H^+^ efflux may be mediated by Na^+^,H^+^-exchangers (NHEs) in the apical membrane. Indeed, our results support the participation of NHEs in H^+^ secretion although the specific isoform(s) involved were not unambiguously determined. We found the apical H^+^ flux in strial marginal cells was reduced by several inhibitors of NHEs, including amiloride, dimethylamiloride (DMA), S3226 and Hoe694 (Fig. 4). The amiloride analogs employed here are more specific for the NHEs than is amiloride itself. Amiloride has additional inhibitory properties against the epithelial sodium channel (ENaC) and Na^+^,Ca^2+^-exchangers [29]. Dose–response curves for DMA and S3226 were fitted by the Michaelis-Menton equation for two drug targets that contributed 18 % (high affinity) and 82 % (low affinity) of H^+^ flux. The high affinity target had an IC_50_ of 3 × 10^−7^ for DMA and 3 × 10^−6^ for S3226. The low affinity targets were taken to be in metabolic pathways rather than membrane transporters; interference with metabolism was shown to decrease the apical H^+^ flux (e.g., removal of glucose, above). High concentrations of drugs (e.g., >100 μM) that are in equilibrium with a lipophilic chemical form (e.g., DIOA and NPPB) [30, 31], are able to integrate into mitochondrial membranes and interfere with ATP production.

Studies on NHE function have often been performed in cells which express multiple isoforms of NHE, which complicates the unambiguous identification of the active isoform(s). Other complicating factors include the strong dependence of the IC_50_ of the amiloride analogs on extracellular [Na^+^], making it difficult to compare specific IC_50_ values for specific NHE isoforms in the literature derived under vastly different [Na^+^] [29].

The greater sensitivity to DMA over S3226 found here is consistent with the H^+^ flux being mediated by NHE2 [29]. NHE2 is expressed in other epithelial cells and can occur in either the apical [32] or basolateral membrane [33]. NHE2 mRNA transcripts were present in stria vascularis at age P10, determined by gene array (GEO Data set: GSE10587). Partial inhibition of the H^+^ flux by the NHE3-specific inhibitor S3226 is consistent with NHE3 also mediating a part of the flux. Strong expression of NHE3 has been demonstrated in the apical membrane of strial marginal cells [9].

NHE1 activity has been identified in the basolateral membrane of vestibular dark cells (which are analogs of strial marginal cells in the vestibular labyrinth) [12], and transcripts of NHE1 were present in stria vascularis at age P10 (GEO Data set: GSE10587), but its contribution, if present, to the measurements here is clearly less than apical NHEs since all inhibitors caused decreases in the net flux. If basolateral NHE1 were the dominant target of any of these inhibitors at the concentrations used, the apical flux would have increased, as during inhibition of basolateral H^+^ efflux via the H^+^,K^+^-ATPase (*vide infra*).

A transient decrease in Isc was observed in stria vascularis with apical perfusion of 500 μM amiloride [12]. The effect was smaller than seen with basolateral perfusion and previously interpreted as only leakage or diffusion to the basolateral side. However, the effect could be a direct inhibition of apical NHE and consequent cellular acidification via inhibited apical NHE activity, consistent with the present results.

NHE6 and NHE9 mRNA transcripts were also present in stria vascularis (GEO Data set: GSE10587). These isoforms occur predominantly in intracellular organelles, but have been observed occasionally in the plasma membrane of some cells [34].

### Significance of NHE in apical membrane

The H^+^ efflux observed in the present experiments in vitro were conducted under conditions that reveal and emphasize the presence of this transporter. That is, the difference in [Na^+^] between the bath and marginal cell cytosol (Table 1) is sufficient to drive H^+^ out of the cell, leading to the observed substantial H^+^ secretion and to Na^+^ absorption. However under normal in vivo conditions, the Na^+^ difference is likely reversed in direction and larger than the outward H^+^ difference (Table 1), assuming reasonable values for cytosolic pH and [Na^+^].

**Table 1:** Estimated Na^+^ and H^+^ gradients across apical membrane of strial marginal cells

	[H^+^],e-b (nM)	[H^+^],c (nM)	[Na^+^],e-b (mM)	[Na^+^],c (mM)	R_H_ (c/e-b)	R_Na_ (e-b/c)	Direction of transport
In vitro	34 (pH 7.4)	63 (pH 7.2)	150	10	1.85	15	Na^+^ in H^+^ out
In vivo	32 (pH 7.5)	63 (pH 7.2)	1	10	1.95	0.1	Na^+^ out H^+^ in

[H^+^], hydrogen ion concentration; [Na^+^], sodium ion concentration; e-b, endolymph (in vivo) or bath (in vitro); c, cytosol; RH RNa, Ratio of H^+^ and Na^+^ concentrations between compartments; Direction of transport into or out of the cell across the apical membrane of strial marginal cells

The cytosolic values in Table 1 are taken from the accepted normative values in the literature that have been utilized for similar calculations on directions of transport in mammalian cells, for example cardiac cell Na^+^,Ca^2+^ exchange and Na^+^,H^+^ exchange [35]. Even though there were some early estimates of intracellular Na^+^ concentration in stria vascularis that were greater [36], a biochemical assay for Na^+^,K^+^-ATPase activity versus Na^+^ concentration demonstrated a peak in activity at 10 mM [37], consistent with physiological control set to a normal level of 10 mM or less.

Therefore under normal homeostatic conditions, this apical membrane exchanger may actually provide a counterbalance to the primary Na^+^ absorptive roles of Reissner’s membrane, Claudius cells and outer sulcus cells [38–43]. Analogous push/pull transport systems of ion homeostasis for K^+^ [17, 43] and for Ca^2+^ [2, 44–46] in endolymph have also been demonstrated. Although not highly active under normative conditions, the apical NHE is poised to respond to pathological increases in endolymphatic [Na^+^], such as during vascular accidents that cause local ischemic anoxia [47, 48]. NHE therefore provides a controlled Na^+^ “leak” into endolymph under normal homeostatic conditions and a Na^+^ absorptive pathway during Na^+^ loading of endolymph.

In addition, the H^+^ flux from the lumen to the marginal cell cytosol (equivalent to HCO_3_^−^ secretion) via NHE under normative conditions would augment the HCO_3_^−^ secretory roles of strial spindle cells and spiral prominence/outer sulcus cells via the pendrin HCO_3_^−^ /anion exchange activity [49].

### Strial H^+^ flux is carried by basolateral H^+^,K^+^ ATPase

**Fig. 5:**
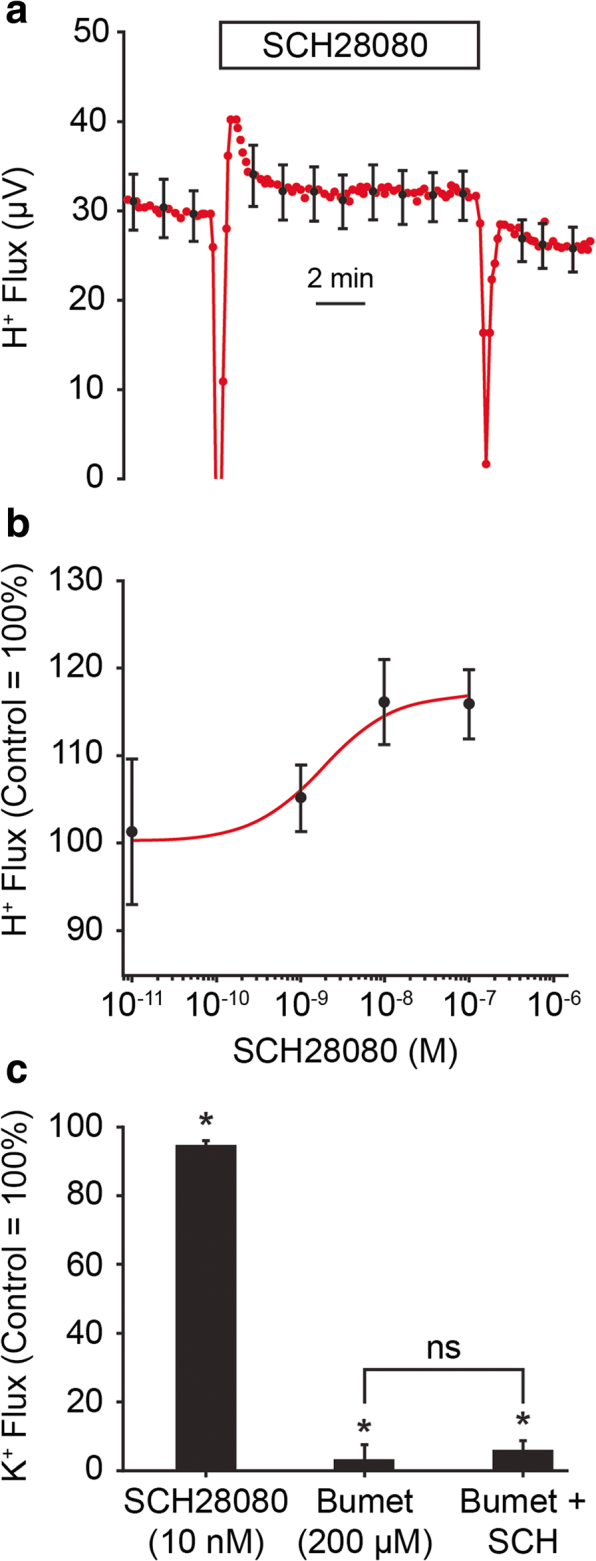
Effects of inhibition of gastric H^+^,K^+^-ATPase on H^+^- and K^+^-fluxes from stria vascularis. **a** Summary traces (*N* = 7) of effect on H^+^ flux of perfusion of SCH28080 (10 nM). Box shows the duration of the experimental period. **b** Concentration-response curve (*N* = 3–7) for stimulation of strial H^+^-flux by SCH28080. Data fit by the Michaelis-Menton equation. **c** Small inhibition of K^+^ flux from stria vascularis by SCH28080 (10 nM; *N* = 7)) and absence of effect (SCH, SCH28080) after inhibition of K^+^ flux by bumetanide (200 μM; *N* = 6; bumet). *, *P* < 0.05; ns, not significant

Gastric H^+^,K^+^-ATPase (consisting of a heterodimer of HKalpha1 and HKbetaG subunits) is expressed in the basolateral membrane of strial marginal cells [8] and its transport function can be inhibited by SCH28080 but not ouabain, in contrast to the colonic H^+^,K^+^-ATPase, which is inhibited by ouabain but completely insensitive to SCH28080 [50]. We found that SCH28080 increased apical H^+^ flux from strial marginal cells (Fig. 5).

The measurements were made over a concentration range of 10^−11^ to 10^−7^ M (*N* = 3–7), with maximal effect of 17.1 % increase and the EC_50_ of SCH28080 was 1.9 × 10^−9^ M. This exquisite sensitivity is about 2 orders of magnitude better than for inhibition of this ATPase in isolated gastric glands, while the IC_50_ of the ATPase by the protonated species of SCH28080 is about 1.5 × 10^−8^ M [51, 52], which is consistent with specific inhibition of this ion pump. By contrast, it was found by Shibata et al that a full decrease of endocochlear potential from vascular or perilymphatic perfusion of SCH28080 occurred at 3 and at 1 mM, respectively [8]. Our observed increase in H^+^ flux suggests that blocking basolateral H^+^ efflux from the marginal cells re-directed the flux through the apical membrane. An increased apical flux during SCH28080 perfusion also implies an elevated cytosolic [H^+^] to drive this transport.

### Strial K^+^ flux is NOT carried by basolateral H^+^,K^+^ ATPase

It was proposed that the basolateral H^+^,K^+^-ATPase contributes to transepithelial K^+^ secretion by taking up K^+^ in parallel to the recognized action of the basolateral Na^+^,2Cl^−^,K^+^ cotransporter [8]. SCH28080 at a virtually maximal dose (10^−8^ M) decreased the apical K^+^ flux in strial marginal cells by less than 5 % (Fig. 5). In order to determine whether this small decrease was physiologically relevant or if it only represented a small but consistent drift in that series of experiments, the sensitivity of this test was increased by first reducing the apical K^+^ flux with a maximal dose (200 μM) of bumetanide. The residual K^+^ flux in the presence of bumetanide was not significantly greater than zero and there was no significant difference by addition of SCH28080 (10^−8^ M). These data demonstrate that H^+^,K^+^-ATPase contributes no more than a minuscule fraction to K^+^ secretion and that H^+^,K^+^-ATPase therefore does not provide an alternative route in the basolateral membrane for K^+^ uptake to the normative route via the Na^+^,2Cl^−^,K^+^ cotransporter.

The report of a decrease in endocochlear potential in response to SCH28080 was ascribed to an inhibition of basolateral H^+^,K^+^-ATPase and a putative drop in basolateral K^+^ uptake into strial marginal cells and consequently a drop in electrogenic K^+^ transport [8]. The present results argue against this interpretation. It is also unlikely that the effect on EP is mediated by changes in marginal cell cytosolic pH or extracellular pH. As described above, the marginal cells possess both apical and basolateral NHEs that could respond to increased cytosolic acid and the KCNQ1/KCNE1 K^+^ channel in the apical membrane of these cells is relatively insensitive to cytosolic acidification in the physiological range [53]. In addition, the KCNJ10 K^+^ channel of the neighboring intermediate cells is also relatively insensitive to changes in extracellular pH [54].

## Conclusions

**Fig. 6:**
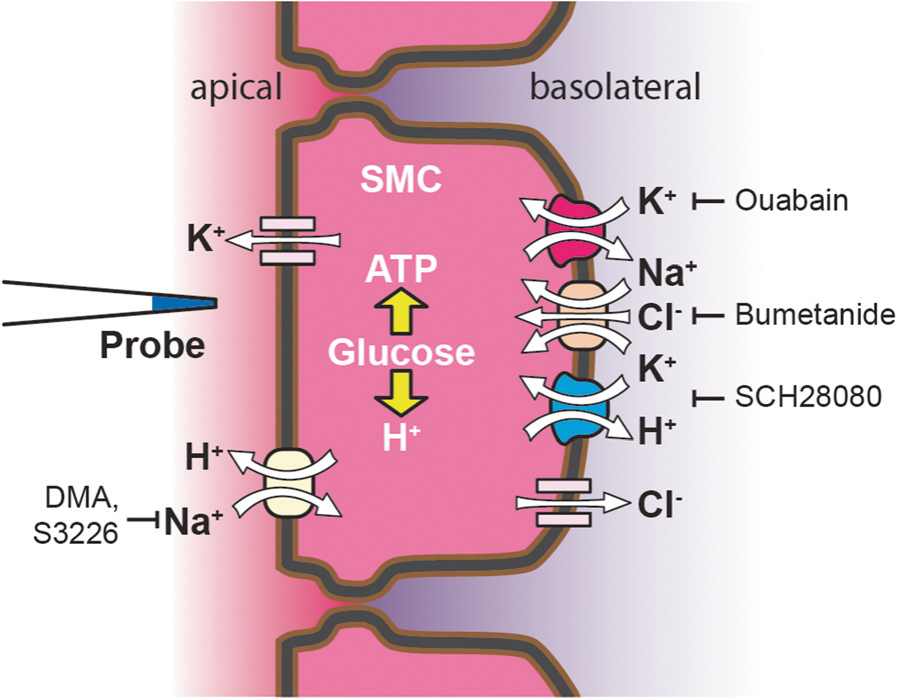
Cell model of H^+^ and K^+^ secretion by strial marginal cells in the cochlea. Transepithelial secretion of K^+^ is mediated by uptake of K^+^ from the basolateral side of the strial marginal cells (SMC) via the Na^+^,K^+^-ATPase and the Na^+^,2Cl^−^,K^+^-cotransporter (NKCC1). The contribution to K^+^ flux by the gastric H^+^,K^+^-ATPase is minimal. K^+^ transport from the cytosol into endolymph is mediated by KCNQ1/KCNE1 K^+^ channels in the apical membrane. Metabolically-generated acid exits the cell via the basolateral gastric H^+^,K^+^-ATPase and the apical Na^+^,H^+^-exchangers. Apical membrane H^+^ exit is increased when the basolateral H^+^,K^+^-ATPase is inhibited and the H^+^ flux re-routed to the apical membrane. Cl^−^ that was taken up into the cytosol via the Na^+^,2Cl^−^,K^+^-cotransporter is recycled across the basolateral membrane via the ClC-K/barttin Cl^−^ channels. Additional putative acid/base transporters, such as basolateral DIDS-sensitive HCO_3_^−^ efflux via NBCe1, are not shown

We have developed a technique for the recording of transepithelial H^+^ and K^+^ flux during continuous perfusion of isolated inner ear tissues at 37 °C and applied it to the investigation of pH homeostatic mechanisms in the cochlea (Fig. 6). Several long-standing questions about the mechanisms underlying cochlear pH homeostasis were resolved. 1) The basolateral H^+^/K^+^-ATPase of strial marginal cells was earlier posited to contribute importantly to the cellular uptake of K^+^ and the subsequent secretion across the apical membrane. Our measurements did not support that proposition, but did demonstrate its significant contribution to basolateral H^+^ flux and its coordination with apical H^+^ flux. 2) The vH^+^-ATPase previously found near the apical membrane of strial marginal cells was posited to contribute importantly to apical H^+^ secretion . Our measurements did not support that proposition. 3) Apical membranes of strial marginal cells were previously shown to express the Na^+^,H^+^ exchanger NHE3 and stria vascularis also expressed mRNA of NHE1, NHE2, NHE6 and NHE9. Our measurements support a functional H^+^ flux from one or more apical Na^+^,H^+^ exchangers. These results advance our understanding of inner ear acid–base balance and provide a stronger basis to interpret the etiology of genetic and pharmacologic cochlear dysfunctions that are influenced by endolymphatic pH.

## Abbreviations

DIDS, 4,4’-Diisothiocyanatostilbene-2,2’-disulfonic acid; DMA, dimethylamiloride; DMSO, dimethylsulfoxide; HOE694, (3-methylsulfonyl-4-piperidinobenzoyl) guanidine methanesulfonate; S3226, (3-[2-(3-guanidino-2-methyl-3-oxo-propenyl)-5-methy]-N-isopropylidene-2-methyl-acrylamide

## Additional file

Additional file 1: “H and K flux raw data”. This file contains the digital ion-selective probe data points collected and analyzed in the text and figures. (XLSX 148 kb)
